# Vitamin D and Weight Cycling: Impact on Injury, Illness, and Inflammation in Collegiate Wrestlers

**DOI:** 10.3390/nu8120775

**Published:** 2016-11-30

**Authors:** Jacqueline N. Barcal, Joi T. Thomas, Bruce W. Hollis, Kathy J. Austin, Brenda M. Alexander, D. Enette Larson-Meyer

**Affiliations:** 1 Department of Family and Consumer Sciences, University of Wyoming, 1000 E. University Avenue, Laramie, WY 82071, USA; jbarcal@uwyo.edu; 2 Department of Athletics, University of Wyoming, 1000 E. University Avenue, Laramie, WY 82071, USA; thomasjj@uwyo.edu; 3 Dr. Bruce Hollis’ Laboratory at the Medical University of South Carolina, Charleston, SC 29425, USA; hollisb@musc.edu; 4 Department of Animal Sciences; University of Wyoming, 1000 E. University Avenue, Laramie, WY 82071, USA; KathyAus@uwyo.edu (K.J.A.); BAlex@uwyo.edu (B.M.A.)

**Keywords:** vitamin D, wrestling, exercise, athletes, inflammation

## Abstract

This study explored the link between vitamin D status and frequency of skin infections, inflammation, and injury in college wrestlers during an academic year. Methods: Serum 25-hydroxyvitamin D (25(OH)D) (*n* = 19), plasma cytokine (TNF-α, IL-6, IL-10) (*n* = 18) concentrations, and body weight/composition were measured and injury/illness/skin infection data were collected in fall, winter, and spring. Results: In the fall, 74% of wrestlers had vitamin D concentrations <32 ng/mL which increased to 94% in winter and spring. Wrestlers lost an average of 3.4 ± 3.9 kg (*p* < 0.001) during the season with corresponding decreases in fat mass and increases in lean mass (*p* < 0.01). An inverse association between 25(OH)D concentrations and total body mass and body fat percentage was observed at all-time points (*p* < 0.01). Concentrations of cytokines were highly variable among individuals and did not change across time (*p* > 0.05). Correlations between vitamin D status, cytokines, or frequency of illness, injury, or skin infections were not observed. Conclusions: A high prevalence of vitamin D insufficiency (<32 ng/mL) and deficiency (<20 ng/mL) was observed in wrestlers and was associated with higher adiposity. It remains unclear if higher vitamin D status would reduce injury, illness, and skin infection risk.

## 1. Introduction

Increasing evidence has linked low vitamin D status to a variety of health conditions including osteoporosis, cardiovascular disease, diabetes, depression, multiple sclerosis, rheumatoid arthritis and certain types of cancer [1,2,3]. Research has also observed a link between low vitamin D status and increased susceptibility of upper respiratory tract infections (URTI) [4] including influenza and the common cold [4,5]. Even though vitamin D is considered a vitamin, it is unique in that it both acts as a hormone, assisting in regulation of serum calcium, and may be obtained from dietary and endogenously synthesized sources. Endogenous synthesis occurs in the skin with exposure to sufficient ultra violet B (UVB) light [1,6] during peak hours (10 a.m. to 2 p.m.). Vitamin D cannot be made in the winter months at distances greater than 37 degrees north or south [2]. Natural or fortified dietary sources include fatty fish, whole milk, and some brands of yogurt, margarine, fruit juice, and ready-to-eat cereals [2]. Although vitamin D deficiency has been considered a nutritional problem of the past, it has re-emerged as a public health concern. In fact, some researchers believe it is an unrecognized epidemic in adults lacking exposure to adequate sunlight [1].

In athletic populations, vitamin D may be important for optimal health and performance. Vitamin D deficiency has been associated with reduced strength [7], prolonged recovery from surgery [8], altered inflammatory markers [9,10], and increased risk for injury and illness [11,12,13] in both athletes and non-athletes. In athletes, the prevalence of vitamin D deficiency and insufficiency varies by sport, training location, skin color [13], adequate sunlight exposure between 10 a.m. and 2 p.m., is more prevalent among athletes who train indoors versus outdoors [11,14,15], and who have higher body fat percentages [16]. A summary of vitamin D status documented in athletes and active individuals is outlined in Table S1. Vitamin D status is generally lower in the winter and among athletes who train predominantly indoors versus outdoors and who live at higher latitudes (i.e., >37 degrees North or South). In addition, serum concentrations tend to decline between fall and spring, and supplementation may or may not be effective for reaching optimum concentrations depending on dosage [17]. Current studies are inconsistent as to whether or not having higher vitamin D status translates to improved performance measures [18].

Wrestlers, particularly at the college level, are at risk for skin infections from mat and skin-to-skin contact and may be more prone to both compromised immune function and poor vitamin D status due to nutrient restriction from cutting weight [19,20,21], indoor training, and competing during a season of limited sun exposure (October-March) when URTI risk is elevated [11,22]. Although the vitamin D status of collegiate athletes has previously been evaluated, little is known about the status of wrestlers who may be at increased risk for deficiency/insufficiency due to indoor training and chronic dietary restriction and weight cycling. Therefore, the purpose of this study was to assess vitamin D status of male college wrestlers during the academic year and determine if low vitamin D status (i.e., low circulating concentrations of 25-hydroxy vitamin D) was associated with documented incidence of acute illness, including skin infections, and with circulating pro-inflammatory cytokines interleukin-6 (IL-6) and tumor necrosis factor-alpha (TNF-α) and the anti-inflammatory cytokine interleukin-10 (IL-10). A secondary purpose was to explore whether weight and body composition changes further impacted seasonal changes in vitamin D concentration and cytokine concentrations. We hypothesized that wrestlers with suboptimal vitamin D status (25(OH)D <32 ng/mL) would have a higher incidence of acute illness including URTI and skin infections, higher pro-inflammatory markers, and lower anti-inflammatory markers compared to wrestlers with optimal vitamin D status (25(OH)D >40 ng/mL).

## 2. Materials and Methods

### 2.1. Study Design

This study took place during the 2014–2015 college academic year. All University of Wyoming (UW) athletes ≥18 years on UW’s National Collegiate Athletic Association (NCAA) Division I wrestling team (*n* = 25) were invited to participate. Participation, however, was not required and the coaching staff received no information on which wrestlers participated in the study so as to avoid potential bias during training or competition. The study was approved by the UW Institutional Review Board (approval code #20140703JB00477, 3 July 2014) with written, informed consent obtained prior to participation. 

### 2.2. Study Overview

Blood was drawn three times during the academic year (September, January, and April) in accordance with seasonal training and typically occurred in the morning after an overnight fast. Athletes were instructed to avoid exercise for at least 12 h prior to the draw. Height and weight were measured and a short questionnaire, administered at these same time points was used to assess vitamin D intake and lifestyle factors which may impact vitamin D status (i.e., estimated time spent outdoors, sunscreen use, use of tanning beds, etc.) [11]. The vitamin D questionnaire was administered in a private setting and took approximately 10 min to complete [11]. Body composition was analyzed by dual energy X-ray absorptiometry (DXA) and occurred no more than 7 days away from the blood draw. Selected information contained within the athletes’ medical charts, including illness and infection history (i.e., upper respiratory tract infections, gastritis, skin infection, etc.), illness progression, prescribed medications, and supplement use was obtained from medical records documented by the Sports Medicine staff as part of routine care.

### 2.3. Blood Analysis

Blood samples were appropriately post-processed and kept frozen at −20 degrees Celsius until analysis. 25(OH)D concentration was evaluated by Diasorin 25(OH)D radioimmunoassay (Bruce Hollis’ Laboratory, the Medical University of South Carolina, Charleston, SC, USA). Inflammatory markers (TNF-α, IL-6, IL-10) were analyzed via enzyme-linked immunosorbent assay (ELISA) (QIAGEN, Santa Clarita, CA, USA) according to manufacturer’s instructions. For cytokine analysis, plasma samples were thawed immediately before being assayed and vortexed for approximately 5 s to ensure adequate mixing. Absorbance was read at 450 nm using a standard ELISA microplate reader. Standards were diluted using a 1:2 dilution series. The eight standards of known cytokine concentrations included in each assay ranged from 15–2000 pg/mL. All samples were analyzed in duplicate. A log transformation was used to generate a regression equation to predict cytokine concentrations, reported as pg/mL.

### 2.4. Body Composition

Body composition was assessed via DXA. Subjects were instructed to avoid food and exercise at least two hours prior to testing. When possible, testing was done first thing in the morning (6:00–9:00 a.m.) after an overnight fast and abstaining from training for at least 12 h. Body mass (kg), fat mass (kg), fat free mass (kg), and body fat percentage were utilized for statistical analysis.

### 2.5. Weight Collection

Weekly weights were obtained from coaching staff from 3 September to 28 February. Wrestlers were weighed on the same scale, with minimal clothing, in their locker room prior to afternoon practice on each Monday by one of the wrestling coaches. In order to calculate the amount of weight lost each week to “make weight” for competition, each athlete’s weight class was subtracted from his recorded Monday weight with the assumption that he would have to lose that amount in order to compete at the end of the week (Friday/Saturday). Because heavyweights are less likely to partake in traditional weight cutting practices, heavyweights were omitted from statistical analyses addressing weekly and seasonal weight changes. These weights were also utilized to calculate average weekly weight changes (Monday to Monday).

### 2.6. Injury, Illness, and Skin Infection

As part of routine medical care, injury, illness, and skin infection data were collected by the team physician and documented in student-athlete medical charts. In addition to documentation done by the physician, a certified athletic trainer (ATC) assigned to the sport performed daily skin checks and was present at each training session. The daily skin check allowed wrestlers to report any unusual formations on the skin.

### 2.7. Statistical Procedures

A sample size calculation for a descriptive study of a continuous variable (i.e., 25(OH)D concentration) was conducted using the average standard deviation for college athletes participating in indoor sports during the fall (8.2 ng/mL) and winter (5.6 ng/mL) from pervious data [11]. Using a 95% confidence interval (CI), an *n* = 19 to 41 was estimated for a total desired CI width of 5 and a sample of 10 to 21 was needed for a total CI width of 7 [23]. Correlation sample calculations for previously identified associations between 25(OH)D and fat mass (*r* = −0.42) [16], frequency of URTI (*r* = −0.40) [11] and TNF-α concentration (*r* = −0.63) [24] using previous coefficients as the expected correlation coefficient (one-tailed *α* = 0.05 and *β* = 0.20) estimated that a sample size of *n* = 14 to 37 would be needed to determine whether correlation coefficients differed from zero. Given these calculations, efforts were placed on recruitment and longitudinal retention of as many of our total population of wrestlers of *n* = 25. 

Data were analyzed using IBM SPSS Statistics 23 software. Repeated measures analysis of variance (ANOVA) was utilized to assess the change in body weight/body composition, serum 25(OH)D, and plasma cytokine concentrations over time. Correlation coefficients (Pearson’s) were used to evaluate the associations among 25(OH)D and body weight/adiposity, weight change during the season, dietary and supplemental vitamin D intake (including vitamin D and calcium), frequency of infection and illness, and cytokine concentrations. Additional multilinear regression modeling was used to evaluate the association between vitamin D status and body weight/body composition adjusting for weight cycling pattern, weight class, age, athletic eligibility year, and other significant predictors (based on analysis of simple correlation coefficients). Spearman’s rank correlations were used to identify intra-cytokine association and association with vitamin D, body composition, illness, injury, and skin infection prevalence. 

## 3. Results

Twenty male wrestlers initially volunteered to participate in the study; nineteen wrestlers reported to baseline testing. All 19 initial participants completed baseline vitamin D testing (September), but body composition data could not be obtained on one athlete due to scheduling conflicts. Baseline characteristics for the 18 wrestlers for which body composition was available are summarized in Table 1.

By the winter data collection point (January), three participants discontinued participation for undisclosed reasons. Complete longitudinal data for vitamin D was therefore available on 16 wrestlers for winter and spring collection and logitudinal body composition and cytokine data were only available on 15 due to difficulty with a blood draw in the fall (*n* = 1) and scheduling conflicts with the DXA measurement (*n* = 1).

During the 2014–2015 academic season, the team as a whole participated in 5 tournaments, 15 dual meets, and six wrestlers competed in the NCAA Division I Wrestling Championships. Starters wrestled an average of 31 matches. Among the athletes who completed the study, there was representation from each of the ten weight classes and each class weight class included a conference dual starter with the exception of the 141 lb weight class in which only fall data was available. 

### 3.1. Vitamin D Status

**Figure 1:**
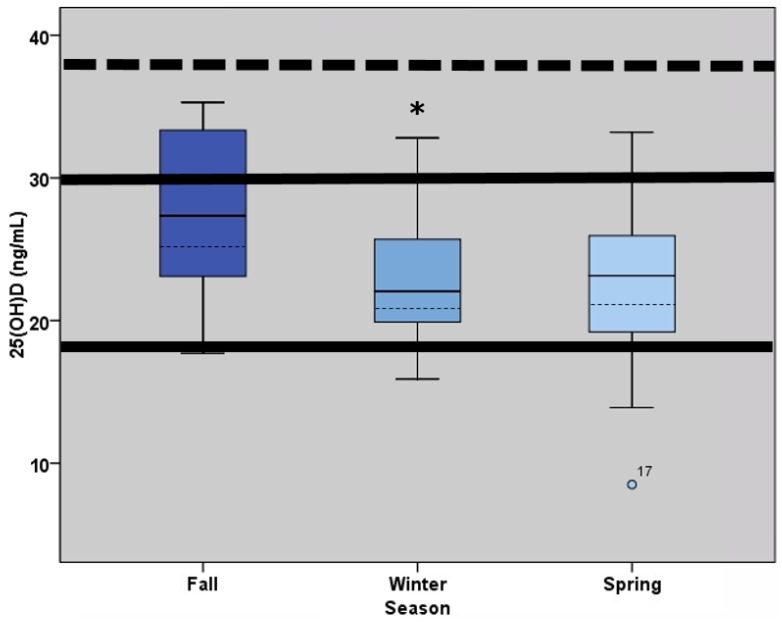
Box plots illustrating the distribution of 25(OH)D concentration (ng/mL) in the fall (*n* = 19), winter (*n* = 16), and spring (*n* = 16). Box extents indicate the 25th and 75th percentile, with the median indicated by a solid dark line and the mean indicated by a dashed line. Central vertical lines (whiskers) extend up to 1.5 interquartile ranges from the end of the box. A circle marks individual points outside of the whiskers indicating a value between 1.5 and 3.0 interquartile ranges of the box. 25(OH)D concentrations <20 ng/mL are considered deficient, concentrations between 20 and 32 ng/mL are considered insufficient (solid horizontal lines), and concentrations >40 ng/mL are considered optimal. * Significant decrease in 25(OH)D observed between fall and winter (*p* < 0.001), reported as mean ± SD.

As shown in Figure 1 below, serum 25(OH)D changed across time (*p* < 0.001) and was highest in the fall and lowest in the winter. In the fall (*n* = 19), five athletes (26.3%) had sufficient vitamin D status whereas twelve (63.2%) and two (10.5%) presented with insufficient (20–32 ng/mL) and deficient status (<20 ng/mL), respectively. In the winter and spring (*n* = 16), one athlete (6%) had sufficient status, and eleven (69%) and four (25%) had insufficient and deficient status, respectively. None of the athletes experienced optimal 25(OH)D status (>40 ng/mL) [5,11] at any time point across the season.

### 3.2. Body Weight and Body Composition

Body weight changes over the course of the training season/academic year (September–May) ranged from −12.0 kg to +4.5 kg with an average season weight change of −3.4 ± 3.8 kg (*n* = 16, *p* = 0.003). Most of the weight loss, averaging 4.1 ± 2.4 kg (−1.0 kg to −9.5 kg), occurred between the fall and winter. This was followed by an average weight gain of 0.21 ± 2.4 kg (−6.0 ± 4.1 kg) per wrestler in between winter and spring. The trend for weight gain between winter and spring remained when omitting the four heavy weight wrestlers in the group (0.06 ± 0.12 kg), but was not apparent when accounting for the nine conference season starters (0.003 ± 0.171 kg). Among the entire group for which weekly weight changes were available (*n* = 19), mean week-to-week weight change (Monday to Monday) averaged −0.19 ± 0.10 kg (−0.37 kg to −0.06) over the course of the study. This minimal change in weekly weight became even smaller when accounting for season starters only (*n* = 10) and also when omitting heavyweights (*n* = 15). Despite minimal changes in weight between team weekly weigh-ins, the amount of weight lost each week to make competition weight, not including heavy weights (*n* = 15), averaged 4.6 ± 1.0 kg (6.3% ± 1.5%). Similar results were observed when accounting for starters. Average weekly weight change did not change across any time point (fall to winter, winter to spring, or fall to spring, *p* > 0.05).

As summarized in Table 2, body composition changed across the training season/academic year (*n* = 15, *p* < 0.01) despite the minimal week-to-week variability in body weight. Absolute total body mass, body mass index (BMI), fat mass, and body fat percentage decreased whereas lean body mass and bone mineral density (BMD) increased.

### 3.3. Cytokine Concentrations and Inter-Cytokine Relationship

As shown in Table 3, there was considerable variability in the concentrations of the various cytokines among individuals and across the academic year (*n* = 15). Concentrations of IL-6 and TNF-α were numerically higher in the fall and spring and lowest in winter whereas concentrations of IL-10 demonstrated the opposite pattern. These slight alterations, however, were not different across time (*p* > 0.05).

In addition, cytokine concentrations were highly inter-correlated at all time points (Pearson’s). IL-6 was positively correlated with both IL-10 and TNF-α during the fall (*n* = 18), winter (*n* = 15), and spring (*n* = 15) (IL-10: *r* = 0.924, *r* = 0.856, and *r* = 0.846, *p* < 0.001, respectively; TNF-α: *r* = 0.950, *r* = 0.837, and *r* = 0.945, *p* < 0.001, respectively). IL-10 also positively correlated with TNF-α at all time points (*r* = 0.897, *r* = 0.767, and *r* = 0.969, *p* < 0.001, respectively).

### 3.4. Illness, Injury, and Skin Infection

Skin infection occurrence over the course of the season (September–May) averaged 1.8 ± 1.3 per participant with fifteen (79%) subjects experiencing at least one skin infection by the end of fall with four (11%) new infections being documented between winter and spring. A total of 35 skin infections were reported over the course of the season with a majority (*n* = 31, 89%) being reported between the fall and winter. Herpes (*n* = 19), ringworm (*n* = 4), and impetigo (*n* = 2) were the most common diagnoses between fall and winter while only herpes (*n* = 4) was reported between the winter and spring. The forehead was the most common skin infection site (*n* = 8) followed by the ear, back, arm/wrist, and lip being the second most frequent location (*n* = 4). 

Eight subjects experienced a documented illness over the course of the study with one subject contracting two illnesses. Six of these were reported between the fall and winter and three were reported between winter and spring. The primary diagnosis was URTI (*n* = 8) which included cough, congestion, and sinus infection. 

There were a total of nine injuries reported in seven different athletes over the year with an average of 0.5 ± 0.7 injuries per wrestler. Collectively, there were 50 total skin infections, illnesses, and injuries reported over the year averaging 2.6 ± 1.5 per wrestler (*n* = 19).

### 3.5. Vitamin D

#### 3.5.1. Vitamin D Intake and Relation to Vitamin D Status

Intake of vitamin D from food alone or food plus supplements is summarized in Table 4. Athletes did not meet the recommended dietary allowance (RDA) for vitamin D intake from food alone (600 IU) at any time point during the year. When supplements were included, seven athletes met the RDA in the fall, five in the winter, and four in the spring. There were no significant changes in dietary vitamin D intake from food alone or food plus supplements across time (*n* = 16, *p* = 0.25 and *p* = 0.707, respectively).

Vitamin D intake from food alone or food plus supplements was not associated with serum 25(OH)D concentrations at any time point (*p* ≥ 0.05). However, in the spring there was a positive association between both total vitamin D intake and vitamin D intake from supplements and vitamin D category (i.e., deficient, insufficient, etc.) such that those with higher overall vitamin D intake had higher categorical status (*n* = 16, *r* = 0.563, *p* = 0.02 and *r* = 0.556, *p* = 0.02, respectively).

#### 3.5.2. Relation between Vitamin D Status and Body Composition

**Figure 2:**
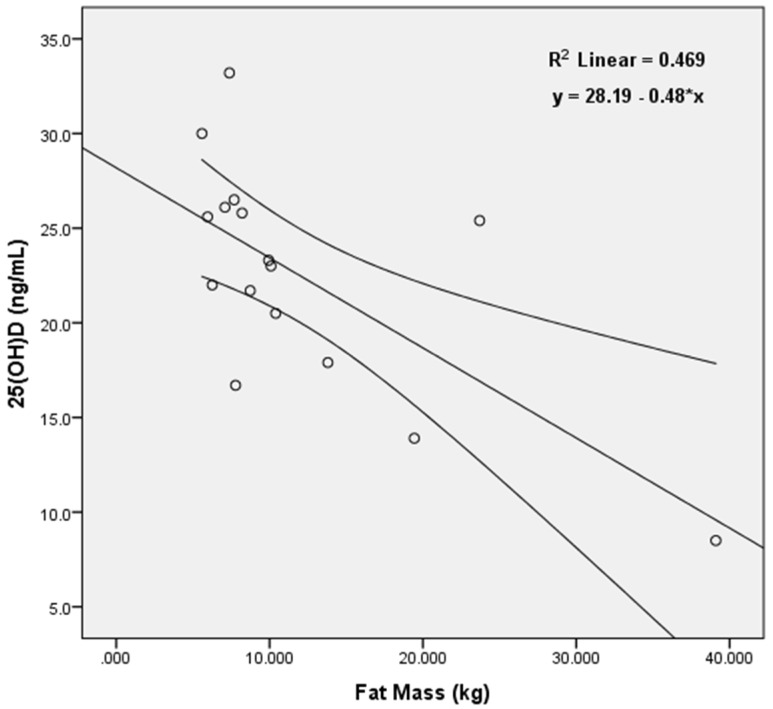
Linear regression model illustrating the association between 25(OH)D and fat mass (kg) in the spring (*n* = 16). Negative associations between these two variables were also observed in the fall and winter.

25(OH)D was negatively correlated with body fat percentage in the fall (*n* = 18, *r* = −0.481, *p* = 0.043,), winter (*n* = 16, *r* = −0.521, *p* = 0.038), and spring (*n* = 16, *r* = −0.565, *p* = 0.18). 25(OH)D was correlated with fat mass in the spring only (*n* = 16, *r* = −0.652, *p* = 0.005) as shown in Figure 2, with a trend for a similar association in the winter (*n* = 16, *r* = −0.446, *p* = 0.083), but not the fall (*n* = 18, *r* = −0.385, *p* = 0.115). 25(OH)D, however, was not associated with total body mass, lean mass, or BMI at any time point (*p* > 0.05). There was an interaction with vitamin D category (sufficient, insufficient, and deficient) and total body mass (*p* = 0.012), fat mass (*p* = 0.001), and body fat percentage (*p* = 0.001) such that those with higher weight and adiposity had lower status. The change in vitamin D status from fall to winter and from winter to spring, however, did not appear to be associated with the change in total body mass or fat mass over these same time points (*p* > 0.05). However, average weekly weight fluctuations (Monday to Monday) from fall to spring positively correlated with spring 25(OH)D (*n* = 16, *r* = 0.663, *p* = 0.005,) such that those with larger average weekly weight changes had higher vitamin D status in the spring.

#### 3.5.3. Relation between Vitamin D Status and Cytokines

Neither 25(OH)D or vitamin D category (sufficient, insufficient, and deficient) correlated with IL-6, IL-10, or TNF-α at any time point (*n* = 15, *p* > 0.05, Pearson’s correlation).

#### 3.5.4. Relation between Vitamin D Status and Illness, Injury, and Skin Infections

Neither 25(OH)D or vitamin D category (sufficient, insufficient, and deficient) was associated with number of illnesses or skin infections at any time point or over the course of the academic year (*n* = 15, *p* > 0.05), however, winter vitamin D category was positively associated with number of injuries incurred from fall to winter such that those with higher status experienced more injuries (*r* = 0.550, *p* = 0.020). 

### 3.6. Cytokines

#### 3.6.1. Relation between Body Composition and Cytokines

At no time point did body composition (total body mass, body fat percentage, fat mass, lean mass, or BMD) influence IL-6, IL-10, or TNF-α over time (*p* > 0.05). Fall and winter fat mass influenced the change in TNF-α over time that approached statistical significance (*n* = 15, *p* = 0.62 × time effect, *p* = 0.73 × time effect).

#### 3.6.2. Relation between Weight Loss and Cytokines

Average weekly weight fluctuations over the course of the year (fall to spring) and cytokine concentrations were not correlated at any time point (*p* > 0.05). Significant correlations were found between weight change from fall to winter and winter cytokine concentrations (*n* = 15, Table 5).

#### 3.6.3. Relation between Cytokine Concentrations and Prevalence of Illness, Injury, and Skin Infection

Cytokine concentrations were not associated with the prevalence of illness, injury, or skin infections at any time point during the study (*n* = 15, *p* > 0.05). 

## 4. Discussion

This study evaluated the vitamin D status of male collegiate wrestlers over the course of an academic year and aimed to determine whether vitamin D status was influenced by vitamin D intake and body composition. We also aimed to determine whether vitamin D status influenced cytokine concentrations, injury, illness, and skin infection incidence. Furthermore, it was evaluated whether wrestlers who “cut” more weight over the course of the season, experienced greater season-induced changes in vitamin D status and/or cytokine concentrations. We found a high prevalence of vitamin D insufficiency and deficiency in wrestlers throughout the academic year, which ranged from 74% in the fall to 94% in the winter and spring, that was inversely associated with body fat percentage and was influenced by week-to-week weight fluctuations, but was not impacted by intake or overall weight loss throughout the season. An association between serum 25(OH)D concentration and risk of injury, infection or illness was not observed. 

Despite an initially high prevalence of vitamin D insufficiency and deficiency in the fall, the status of college wrestlers declined significantly across the academic year as has been previously shown in other athlete groups [11,25,26,27,28,29,30]. The majority of the decline occurred between the fall (September) and winter (January) with the incidence of insufficiency/deficiency reaching 94% in the winter and spring. The overall prevalence of suboptimal status was higher than previously reported in our laboratory in college athletes from mixed sports [11,24] and those of other indoor athletes [26,27] and sportsmen training near the equator where suspected sun avoidance was the culture [31]. Although it was not surprising that wrestlers had low vitamin D status in the winter and spring due to their exclusive indoor training regimen, it was somewhat surprising that status was so low in the fall when wrestlers engaged in close to eight weeks of outdoor training typically between 2:00 and 5:00 p.m. in mostly sunny conditions at an elevation of between ~7200 and 8400 feet [25]. The higher than expected prevalence of insufficiency and deficiency may be a combination of late afternoon training, which misses peak hours of 10:00 a.m.–2:00 p.m., clothing worn, or the higher body fat percentage of some of the athletes [16] when weekly weigh-ins are not required. In agreement with previous studies in both athletes and non-athletes [16,30,32,33,34], body adiposity was negatively associated with serum 25(OH)D concentration across the academic year. Although the specific mechanism for this association is not fully understood, the lipophilic properties of vitamin D are thought to allow sequestration in adipose tissue, which thereby decreases circulating 25(OH)D concentrations [30,35].

The overall low vitamin D intake may have also contributed to low status. Vitamin D intake was, on average, less than 50% of the RDA across the season, which is consistent with research in other athletic populations [11,25,26,36]. Vitamin D supplementation was also low. For instance, depending on the time of the year, only two to three wrestlers reported taking a vitamin D supplement and only five to six reported taking a multi-vitamin. In agreement with previous studies [11,32], vitamin D intake from food or supplements was not directly associated with vitamin D status. Ironically, in the current study the wrestler with the lowest serum 25(OH)D concentration had the highest body mass, the third highest body fat percentage, and reported taking a vitamin D supplement during the entire academic year. He also experienced a significant decline in body fat percentage (37.7% in the fall to 19.6% in the spring). This suggests that the supplemental dose taken (not reported) was not sufficient to counteract the negative influence of adiposity on status across the season despite a significant reduction in body adiposity. 

Unique to this study was our evaluation of whether vitamin D status impacted cytokine concentrations over the course of the year. Our initial hypothesis was that athletes with lower vitamin D status would have higher pro-inflammatory cytokines and lower anti-inflammatory cytokines than those with higher status. Our lack of an association between serum 25(OH)D and cytokine concentrations during the season, however, may be explained by the overall low vitamin D status of our wrestlers and/or the high variability in cytokine concentrations throughout the year. Although all blood cytokine concentrations were obtained after at least 12 h of physical inactivity, approximately eight weeks (August–September) of conditioning (6 days/week) and strength training (3 days/week) occurred before the initial data collection in September. Thus, preseason training may have elevated cytokines in some or all athletes. It is not well established how a single bout of exercise or regular training influences cytokine concentrations, or how long athletes should refrain from exercise to reveal cytokine samples reflective of overall health. For example, some research has shown that after a single bout of intense physical activity, cytokine concentrations return to baseline within 2–3 h after exercise terminates [9,37] while others suggest that cytokines remain elevated even 24 h following exercise [38,39]. 

In the present study, IL-6 and TNF-α concentrations decreased between fall and winter. This was followed by an increase back toward the fall baseline between winter and spring. IL-10 concentration, in contrast, increased between the fall and winter also returning toward baseline in the spring. The decrease in TNF-α concentration between the fall and winter was somewhat unexpected due to intense practices and weight training sessions 5–6 days per week, which would be expected to induce a low level inflammatory response. Perhaps, TNF-α was not elevated due to a concurrent rise in IL-10 concentrations. IL-10 is a potent anti-inflammatory cytokine that has been shown to assist in the down-regulation of TNF-α during exercise [37,40]. In agreement with our findings, previous research has also shown a lack of correlation between 25(OH)D and TNF-α [32,41,42,43,44,45]. Our lack of a relationship between vitamin D status and TNF-α concentration, however, is contradictory to Willis et al. [24], and others who have found a significant inverse relationship between 25(OH)D and TNF-α concentrations in both humans and mice [45,46,47,48].

In the current study, the majority of skin infections were reported between the fall and winter (*n* = 31) as compared to between winter to spring (*n* = 4). This was somewhat expected, as wrestlers transition from summer conditioning and strength training to training in the wrestling room where skin-to-skin and skin-to-mat contact are more frequent. This time period also coincides with the suspected reduction in cutaneous vitamin D synthesis (from limited sun exposure and reduced synthesis capacity) and frequent dietary restriction as wrestlers work to maintain competition weight on a week-to-week basis. As previously noted, the majority of the decrease in serum 25(OH)D concentration occurred between fall and winter (28.2 ± 5.2 vs. 22.8 ± 4.6 ng/mL), however, vitamin D intake remained unchanged despite the suspected intentional energy restriction, at least in some wrestlers. We had hypothesized, however, that the increased skin infection risk would be associated with low serum 25(OH)D. The hormonally active form of vitamin D, 1,25(OH)_2_D_3_, has been shown to up-regulate expression of anti-microbial peptides (AMP’s) and assist in the defense against bacterial infection [49]. Our lack of association between vitamin D status and frequency of both skin and other documented illnesses, nevertheless, may be due to the overall high frequency of vitamin D insufficiency and deficiency. This precluded maintenance of 25(OH)D concentrations in a range not high enough to experience a beneficial effect. For example, when Halliday et al. evaluated the relationship between vitamin D status and illness in collegiate athletes from our same university, athletes with higher 25(OH)D concentration in the winter and spring were found to experience fewer illnesses over the course of the year [11]. Reduction in illness frequency, however, was not apparent until serum 25(OH)D concentrations approached approximately 40 ng/mL [11]. In the current study, only one wrestler maintained vitamin D status in the sufficient category (>32 ng/mL) in both winter and spring and none maintained 25(OH)D concentration greater than 40 ng/mL. Future research should include a vitamin D supplementation trial that elevates concentrations of 25(OH)D above 40 ng/mL to allow for a better analysis of the impact of maintaining optimal vitamin D status on inflammation, illness, and skin infection in wrestlers.

The present study did not reveal a consistent relationship between vitamin D status and number of injuries sustained. This may be partially explained by our low injury rate, injury underreporting, or small sample size. In the present study, our injury frequency averaged approximately 0.5 injuries per wrestler which was lower than previous reports in male ballet dancers (1.9 injuries/dancer) [50], Taiwanese elite wrestlers (4.2 injuries/wrestler) [51], and high school wrestlers (5.2 injuries/wrestler) [52]. With the identification of vitamin D receptors in skeletal muscle cells [53], it is not surprising that several previous studies have found associations between higher vitamin D status and fewer incidences of injuries, including in professional American football athletes [54,55]. Furthermore, vitamin D supplementation in ballet dancers has been shown to reduce injuries over a 4-month period when compared to no supplementation [7]. Other research has indicated that while maintaining higher vitamin D concentrations may not prevent injury, it may assist in expediting recovery [56]. One unexpected result of the current study was the positive correlation between vitamin D status in the winter and the number of injuries that occurred between the fall and winter. It is likely that this association is coincidental due to the small number of injuries and small sample size. Overuse, over-training, and/or poor wrestling technique may also confound our findings. Unfortunately, duration of injury or injury rehabilitation response was not evaluated in the present study.

Collectively, the reported total number of skin infections, illnesses, and injuries averaged 2.7 ± 1.5 per wrestler (*n* = 20), however, actual rates may be higher due to underreporting by the athlete to avoid restrictions from practice and/or competition. The five injuries that occurred in the four wrestlers between fall and winter included a concussion, wrist fracture, meniscus tear, foot sprain, and a shoulder pain complaint due to surgery that occurred at the beginning of the semester.

We found that fat mass at all three time points (fall, winter, and spring) influenced the change in TNF-α across time. This finding is in agreement with previous reports indicating a positive association between adiposity and inflammation [36]. This may warrant special attention to those athletes with higher adiposity, as they may be more prone to injury and illness. Interestingly, a non-significant inverse relationship between all winter cytokine concentrations and the total number of illnesses, injuries, and skin infections was observed, implying that between September and January, a period when training is at its peak, lower cytokine concentrations were associated with increased risk for illness, skin infection or injury. 

In the current study it is important to note that over the course of the season wrestlers lost an average of 3.5 kg (−12.0 to +4.5 kg), which is lower than the 7.9 ± 0.8 kg loss reported by Melby et al. [57]. On average, wrestlers started the training week (Monday) 4.6 ± 1.0 kg (6.3% ± 1.5%) over their weight class suggesting an approximate 5 kg weight loss each week (Monday–Friday) to “make weight” for competition. The weight loss observed in this study is on the lower end of the previously reported weekly loss of 5.0–9.1 kg reported by Steen and Brownell [19], but higher than the 2.3 and 3.4 kg deficits observed by Lakin, Steen, and Oppliger [20] and Lingor and Olson [21], respectively. Furthermore, average weekly weight loss, Monday to Monday, averaged <0.5 kg which is significantly lower than the suggested weight loss between Monday to Friday and lower than any of the aforementioned studies [19,20,21]. No significant differences in average weekly weight loss between starters and non-starters were observed. This may be due to non-starters competing in non-conference dual meets on the weekends requiring them to engage in similar weight management behaviors as starters. Culturally, coaches also implemented a “maximum weight over” policy that prohibited wrestlers from participating in normal practice activities if they reported to Monday training more than 4.6 kg over their weight class. The positive association between average weekly weight change (Monday to Monday) over the course of the season and spring 25(OH)D concentration and status implies that those who had larger weekly weight fluctuations tended to have higher vitamin D by the spring, which is in contrast to our hypothesis. While unexpected, this observation may reflect a release of vitamin D from adipose tissue by those wrestlers who lost more weight each week.

### Limitations

Although our study captured a unique group of athletes potentially at increased risk for poor vitamin D status, it has several limitations. This includes the small sample size, limited racial differences, and the limited variability in 25(OH)D among individual wrestlers at all-time points. For example, a majority of wrestlers in fall, winter, and spring had serum 25 (OH)D concentrations in the deficient and insufficient range with only a few athletes (*n* = 5 in the fall and *n* = 1 in the winter) with concentrations in the sufficient range and none in the optimal range. This precludes comparisons between those with deficient/insufficient status and those sufficient or optimal status, and also limits statistical power. In addition, participants in the current study were closely monitored by a registered dietitian (J.N.B.) which may have positively influenced dietary habits (resulting in better fueling choices, healthier weight loss practices, and lowered post-exercise cytokine responses). The “maximum weight over” policy established by the sport coaches to prevent wrestlers from reporting to Monday practice more than 4.6 kg over their weight class, may have reduced week-to-week weight fluctuations. The low number of skin infections, illnesses, and injuries reported in the current study between the winter and spring may be the result of under-reporting, under-recording, or season-to-season variability of which made detection of relations with vitamin D status difficult. In addition, data collection, particularly for cytokine concentrations, began after approximately eight weeks of fall conditioning (August-September) which may have altered the true picture of how vitamin D status influences such concentrations during training and competition, however, they are a reality for the in-season college athletes. Finally, vitamin D binding protein (VDBP) and gene polymorphisms were not addressed in this study which may have influenced 25(OH)D concentrations [58,59].

## 5. Conclusions

This study is the first, to our knowledge, to directly analyze the relationship between vitamin D status’ and weight cycling’s impact on inflammation, skin infection, illness, and injury in wrestlers. Overall, there was a lack of association between vitamin D and prevalence of illness, injury, and skin infection which was in partial contrast to our hypothesis and supports the need for further research in wrestlers. Future research should attempt to analyze a larger sample size, include a supplementation group, and also include wrestlers from varying latitudes. Although we were not able to shed light on the ability of vitamin D to influence cytokines or reduce risk of illness, injury, or infection, our study revealed that despite the stereotypical extreme weight making practices of wrestlers, this group experienced minimal weekly weight fluctuations to reach their goal weight and this likely contributed to their ability to maintain or gain lean body mass while decreasing body fat over the course of the year. Although most of the wrestlers did not utilize vitamin D supplements, exploring the potential benefits of vitamin D supplementation for reducing frequency of illness, injury, and skin infections should be explored in future research especially in a group that appears to have a high prevalence of insufficiency and deficiency.

## Figures and Tables

**Table 1:** Baseline characteristics of 18 male wrestlers ^1^.

Age (years)	Height (cm)	Weight (kg)	BMI (kg/m^2^)	Body Fat (%)
20.9 ± 2.0	171.8 ± 15.3	87.4 ± 18.6	27.3 ± 4.0	19.0 ± 7.0
19–23	162.6–193.0	62.0–121.7	22.6–35.4	12.5–37.7

^1^ Data reported as mean ± SD with range listed beneath; Caucasian (*n* = 15); Spanish Italian (*n* = 1); Asian (*n* = 2). Abbreviations: BMI, body mass index.

**Table 2:** Body composition across the academic year ^1^.

Measurement	Fall	Winter	Spring	Sig. (*p*) ^2^
Weight (kg)	90.6 ± 18.0	86.5 ± 17.0	86.7 ± 16.0	<0.001
BMI (kg/m^2^)	27.6 ± 3.9	26.4 ± 3.6	26.7 ± 3.4	0.001
Lean Mass (kg)	69.0 ± 10.4	70.8 ± 9.8	70.5 ± 9.8	0.003
Fat Mass (kg)	17.6 ± 10.2	11.8 ± 9.4	12.1 ± 9.1	<0.001
Body Fat (%)	19.4 ± 7.1	13.2 ± 7.4	12.8 ± 5.0	<0.001
BMD (g/cm^2^)	1.42 ± 0.1	1.45 ± 0.1	1.47 ± 0.1	0.001

^1^ Data reported as mean ± SD; ^2^ Repeated measures ANOVA of body composition changes across time (*n* = 15) (statistic × time effect).

**Table 3:** Cytokine concentrations across the academic year ^1^.

Cytokine	Fall	Winter	Spring	Sig. (*p*) ^2^
IL-6 (pg/dL)	110 ± 229	73 ± 144	120 ± 306	0.335
IL-10 (pg/dL)	254 ± 469	362 ± 938	246 ± 559	0.270
TNF-α (pg/dL)	1549 ± 2361	886 ± 1117	1293 ± 2197	0.287

^1^ Data reported as mean ± SD. ^2^ Repeated measures ANOVA of body composition changes across time (*n* = 15) (statistic × time effect).

**Table 4:** Vitamin D intake from food and supplements across the academic year ^1^.

Vitamin D Source	Fall	Winter	Spring
Dietary Intake	257 ± 212	211 ± 135	250 ± 191
Intake from Supplements	443 ± 996	370 ± 569	296 ± 758
Combined Vitamin D Intake	1549 ± 2361	886 ± 1117	1293 ± 2197

^1^ Reported as International Units (IU), mean ± SD (*n* = 16).

**Table 5:** Association between cytokine concentrations in the winter and total body mass change between fall and winter (*n* = 15).

Spearman’s Rank	*Correlation (r)*	Sig. (*p*)
IL-6	0.497	0.060
IL-10	0.561	0.029
TNF-α	0.540	0.038

## Supplementary Materials

The following are available online at http://www.mdpi.com/2072-6643/8/12/775/s1, Table S1: Summary of Vitamin D Status in Athletes and Active Individuals.
