# Supplementary material for: Vitamin D and Weight Cycling: Impact on Injury, Illness, and Inflammation in Collegiate Wrestlers
Source: Nutrients. 2016 Nov 30;8(12):775. doi: 10.3390/nu8120775 (PMC5188430; doi:10.3390/nu8120775)
Supplement: Supplementary file 1 [file nutrients-08-00775-s001.docx]

Vitamin D and Weight Cycling: Impact on Injury, Illness, and Inflammation in Collegiate Wrestlers

Jacqueline N. Barcal, Joi T. Thomas, Bruce W. Hollis, Kathy J. Austin, Brenda M. Alexander and D. Enette Larson-Meyer

**Table S1.** Summary of Vitamin D Status in Athletes and Active Individuals.

| **Author** | **Sport** | **Subjects/Methods Description** | **Season** | **Location** | **Reference Ranges (ng/mL)** | **Average 25(OH)D (ng/mL)** | **Vitamin D Status** | **Observations** |
| --- | --- | --- | --- | --- | --- | --- | --- | --- |
| He et al.,  2016 [1] | Multiple sports | 20 males,  20.1 ± 1.7 years;  80.8 ± 13.1 kg | November | UK (53° N) | Not defined | 21.8 (17.3–28.4) | Not reported | 5000 IU of vitamin D daily increased 25(OH)D concentrations over 14 weeks of supplementation  (*p* = 0.001). |
| Beck et al.,  2015 [2] | Ballet | 47 ballet dancers,  14 ± 1.2 years;  51.4 ± 7.0 kg;  93.6% Caucasian | March–September | NZ (44.1° S) | Sufficient: >30  Insufficient: 20–30  Deficient: <20 | 30.0 ± 7.6 | Sufficient:  91.1% (*n* = 41)  Insufficient:  8.8% (*n* = 4)  Deficient:  0% (*n* = 0) | A majority of dancers had sufficient vitamin D concentrations despite only meeting 45.5% of the RDI for vitamin D. |
| Fitzgerald et al., 2015 [3] | Ice Hockey | 53 male junior and collegiate ice hockey players; 20.1 ± 1.5 years;  84.3 ± 6.9 kg; 12.5% ± 3.9% | May–June | MN (44.9° N) | Sufficient: >32  Insufficient: 20–32  Deficient: <20 | 35.7 ± 8.9 (20.3–65.4) | Sufficient:  62.3% (*n* = 33)  Insufficient:  37.7% (*n* = 20)  Deficient:  0% (*n* = 0) | Fat mass, but not body mass inversely correlated with 25(OH)D (*r* = −0.44). |
| Heller et al.,  2015 [4] | Multiple college sports | 42 Division I colleg  85.0 ± 28.7 kg;  BMI = 25.7 ± 6.1 kg/m^2^ | August–October | Laramie, WY (41.3° N) | Optimal: >40  Sufficient: >32  Insufficient: 20–32  Deficient: <20 | 40.5 ± 15.2 | Optimal:  59.5% (*n* = 25)  Sufficient:  14.3% (*n* = 6)  Insufficient:  14.2% (*n* = 6)  Deficient:  11.9% (*n* = 5) | A majority of athletes (~74%) had sufficient or optimal status. Female athletes tended to have higher fall 25(OH)D concentrations than male athletes. Fat mass was a predictor of 25(OH)D concentration (*p* = 0.01). |

**Table S1.** *Cont.*

| **Author** | **Sport** | **Subjects/Methods Description** | **Season** | **Location** | **Reference Ranges (ng/mL)** | **Average 25(OH)D (ng/mL)** | **Vitamin D Status** | **Observations** |
| --- | --- | --- | --- | --- | --- | --- | --- | --- |
| Maroon et al.,  2015 [5] | Football (NFL) | 80 football athletes  27.4 ± 11.7 years;  Black (*n* = 67),  Caucasian (*n* = 13) | Spring & Summer | Pittsburg, PA (40° N) | Adequate: >32  Insufficient: 20–32  Deficient: <20 | 27.4 ± 11.7 | Sufficient:  31.3% (*n* = 25) Insufficient:  42.5% (*n* = 34)  Deficient:  26.3% (*n* = 21) | Black athletes had lower 25(OH)D than white  (*p* = 0.001). All athletes in the deficient category were black, 91% of insufficient category were black. Number of seasons in NFL was correlated with vitamin D status (*r* = 0.388,  *p* ≤ 0.01). 21% of athletes experienced a bone fracture, 9 participant >1 fracture. No correlation between vitamin D and bone fracture. |
| Farrokhyar et al., 2014 [6] | Review of multiple sports | 23 studies (2008–2014) with 2313 athletes, Male:  (*n* = 1758), Female: (*n* = 555),  22.5 ± 5.0 years | Multiple Seasons | UK/Ireland, Spain/France, Australia, Israel/Mid East. Median Latitude (40° N) | Insufficient: <32 | Not Reported | Insufficient:  56% (*n* = 1295) | 56% of athletes had inadequate vitamin D status, 39% in UK, risk for inadequacy higher in winter and spring than summer/fall seasons. Higher risk of inadequacy for regions  >40° N. |
| Koundourakis et al., 2014 [7] | Soccer | 67 male soccer players 25.6 ± 6.2 years;  77.7 ± 7.1 kg | May | Crete, Greece (35.2° N) | Sufficient: >30  Insufficient: 20–30  Deficient: <20 | 34.4 ± 7.1 | Insufficient:  55.2% (*n* = 37) | Despite significant increases in 25(OH)D concentrations, all performance measures declined. |

**Table S1.** *Cont.*

| **Author** | **Sport** | **Subjects/Methods Description** | **Season** | **Location** | **Reference Ranges (ng/mL)** | **Average 25(OH)D (ng/mL)** | **Vitamin D Status** | **Observations** |
| --- | --- | --- | --- | --- | --- | --- | --- | --- |
| Valtuena et al.,  2014 [8] | Multiple sports | 408 elite athletes;  22.8 ± 8.4 years;  84.3 ± 6.9 kg; 12.5% ± 3.9% | Entire year | Barcelona, Spain (41.4° N) | Sufficient: >30  Insufficient: 20–32  Deficient: <20 | 22.8 ± 9.4 | Sufficient:  18% (*n* = 33)  Insufficient:  37.7% (*n* = 20)  Deficient:  44.3% (*n* = 0) | Even during the summer, 87% of the sample was insufficient. Surprisingly, highest concentrations were observed in the winter. |
| Villacis et al.,  2014 [9] | Multiple Sports | 223 Division I athletes, Male: (*n* = 121),  Female: (*n* = 102),  BMI: 29.2 ± 4.3 kg/m^2^, dark skin (*n* = 94), light (*n* = 129) | Entire Year | University of Southern California (34° N) | Normal: >32  Insufficient: 20–32  Deficient: <20 | Baseline:  40.1 ± 14.9 | Sufficient:  66.4% (*n* = 148)  Insufficient:  30.5% (*n* = 68)  Deficient:  3.1% (*n* = 7) | Men’s and women’s basketball had the highest percentage of abnormal 25(OH)D (46.2%/61.5%, respectively), women’s cross-country (7.1%), women’s lacrosse (0%). 64% of those with dark pigmented skin had abnormal values, 10.8% of light skinned athletes. Male athletes 2.8-fold higher odds of abnormal values when compared  to females. |

**Table S1.** *Cont.*

| **Author** | **Sport** | **Subjects/Methods Description** | **Season** | **Location** | **Reference Ranges (ng/mL)** | **Average 25(OH)D (ng/mL)** | **Vitamin D Status** | **Observations** |
| --- | --- | --- | --- | --- | --- | --- | --- | --- |
| Close et al.,  2013a [10] | Soccer and Jockeys | Study 1: Vitamin D status: 61 male pro athletes,  non-vitamin D supplemented  (18–25 years; 56.1–99.0 kg) + 30 male controls. Study 2: Vitamin D supplementation with (5000 IU/day) in 14 male pro soccer players (EPL) | November–January | UK (53° N) | Optimal: >40  Adequate: >20  Inadequate: 12–20  Deficient: 5–12  Severe Def.: <5 | Not Reported | Athletes (PRE):  Optimal:  2% (*n* = 1)  Adequate:  38% (*n* = 23)  Inadequate:  26% (*n* = 18)  Deficient:  28% (*n* = 17)  Severely Deficient:  7% (*n* = 4) | Only 1/61 athletes and 1/30 control participants had 25(OH)D >40 ng/mL at baseline. Study 2: Sig increase in vitamin D following supplementation  (*p* = 0.0029). No significant increase with placebo. 7/10 participants insufficient at baseline. 2/10 were deficient. Following supplementation, 60% > 40 ng/mL. One supplemented soccer athlete's 25(OH)D went from 22.4 ng/mL to  55.7 ng/mL and saw improved performance in all 6 tests. Vitamin D supplemented group showed significant improvements in 10 m sprint times and  vertical jump. |

**Table S1.** *Cont.*

| **Author** | **Sport** | **Subjects/Methods Description** | **Season** | **Location** | **Reference Ranges (ng/mL)** | **Average 25(OH)D (ng/mL)** | **Vitamin D Status** | **Observations** |
| --- | --- | --- | --- | --- | --- | --- | --- | --- |
| Close, et al.,  2013b [11] | Multiple club sports | 30 club-level athletes,  20–24 years | January–April | UK (53° N) | Optimal: >40  Adequate: >20  Inadequate: 12–20  Deficient: 5–12  Severely Deficient: <5 | 20.4 ± 9.6 | Adequate:  23% (*n* = 7)  Inadequate:  57% (*n* = 17)  Deficient:  20% (*n* = 6) | 6 and 12 weeks of either 20,000 or 40,000 IU vitamin D per week, respectively, increased vitamin D concentrations in all subjects. After supplementation all subjects had concentrations  >20 ng/mL. Increasing vitamin D concentrations did not have any significant effect on performance measures. |
| He et al.,  2013 [12] | Endurance trained during November | 225 F: (*n* = 68),  M: (*n* = 157). 21 ± 3 years; 73.4 ± 11.4 kg;  BMI: 23.5 ± 2.3 kg/m^2^ | Winter | Loughborough University, UK (53° N) | Optimal: >48  Adequate: 20–48  Inadequate: 12–20  Deficient: <12 | Baseline: 23 ng/mL | Optimal:  5% (*n* = 11)  Adequate:  57% (*n* = 128)  Inadequate:  30% (*n* = 68%)  Deficient:  8% (*n* = 18) | Decrease in 25(OH)D from beginning to end  (*p* = 0.003), 70% of athletes experiencing a URTI had a decrease in performance during URTI. Vitamin D sufficient athletes experienced fewer URTI symptoms when compared to deficient athletes. Duration of URTI symptoms sig higher in deficient group when compared to all other groups. |

**Table S1.** *Cont.*

| **Author** | **Sport** | **Subjects/Methods Description** | **Season** | **Location** | **Reference Ranges (ng/mL)** | **Average 25(OH)D (ng/mL)** | **Vitamin D Status** | **Observations** |
| --- | --- | --- | --- | --- | --- | --- | --- | --- |
| Kopec et al.,  2013 [13] | Soccer | 24 Caucasian Polish soccer players; 26 years | September (Post Summer) and April (Post Winter) | Poland/(51° N) 2 weeks in Jan at (30° N) | Normal: 30–60 Insufficient: 21–29 Deficient: <20 *(Endocrine Soc.)* | Post-summer: 30.82 ± 9.04 Post winter: 24.96 ± 9.91 | Sufficient:  Post-summer:  50% (*n* = 12)  Post-winter:  16.7% (*n* = 4)  Insufficient:  Post-summer:  37.5% (*n* = 9)  Post-winter:  45.8% (*n* = 11)  Deficient:  Post-summer:  12.5% (*n* = 3)  Post-winter:  37.5% (*n* = 9) | Post-summer uniform covered 35%,  post-winter covered 80%. Participants did not take any supplements containing vitamin D or calcium. |
| Magee et al,  2013 [14] | Boxers & Paralympians | 74 athletes (33 boxers,  17 Paralympians,  34 Gaelic Athletic Association (GAA) players | November (Boxers/paralympians) or March (GAA) | Ireland (51° N) | Not defined | 19.2  (13.0–25.6) | Deficient:  55% (*n* = 46) | 94% of GAA athletes, 29% of boxers, 27% of Paralympians were deficient (<20 ng/mL). |
| Wilson et al.,  2013 [15] | Jockeys | 36 male jockeys;  Flat: (*n* = 19); 27 ± 5 years; 56.1 ± 2.9 kg;  Jump: 25 ± 5 years;  65.3 ± 2.5 kg | January–April | Liverpool, UK (53° N) | Sufficient: >20 Insufficient: 12–20 Deficient: <12 | Flat: 37.5 ± 28 Jump: 35.1 ± 14 | Sufficient:  22% (*n* = 8)  Insufficient:  31% (*n* = 11) Deficient:  47% (*n* = 17) | Most jockeys (78%) were either deficient or insufficient. |

**Table S1.** *Cont.*

| **Author** | **Sport** | **Subjects/Methods Description** | **Season** | **Location** | **Reference Ranges (ng/mL)** | **Average 25(OH)D (ng/mL)** | **Vitamin D Status** | **Observations** |
| --- | --- | --- | --- | --- | --- | --- | --- | --- |
| Wolman et al.,  2013 [16] | Ballet | 19 ballet dancers,  26 ± 8.9 years;  54.3 ± 10.5 kg | February and August | UK (52° N) | Sufficient: >30 Insufficient: 10–30 Deficient: <10 | Winter: 14.9  Summer: 23.9 | Sufficient:  Winter:  0% (*n* = 0)  Summer:  16% (*n* = 3)  Insufficient: Winter:  74% (*n* = 14)  Summer:  74% (*n* = 14)  Deficient:  Winter:  26% (*n* = 5)  Summer:  10% (*n* = 2) | Average increase in vitamin D was significant, averaging  9 ng/mL (*p* < 0.001). |
| Galan et al.,  2012 [17] | Soccer (Professional) | 26 Caucasian soccer players (26.7 ± 3.6 years; BMI: 23 ± 1.1 kg/m^2^), supplemented with  1000 mg of Ca + 200 IU of vitamin D | Mid October and Early February | Andalusia, Spain (37° N) | Sufficient: >30 Insufficient: 20–30 Deficient: <20 | October:  39.4 ± 9.6  February:  28.0 ± 5.9 | Sufficient:  October:  93% *(n* = 13)  February:  36% (*n* = 10) | October: 93% (*n* = 26) had 25(OH)D >30. February: 64% (*n* = 18) had levels <30. Despite sun exposure, few reached recommended levels in mid-winter and 2/3 had vitamin D deficiency in early February. |
| Peeling et al.,  2012. [18] | Multiple sports; mainly gymnastics, sailing, water polo | 72 elite athletes, Caucasian (*n* = 68), Asian (*n* = 4), Male: (*n* = 43), Female: (*n* = 29) | February (Summer: Avg. temp 31.5 °C, dUVR 12) | Australia (31° S) | Sufficient: >32 Insufficient: 20–32 Deficient: <20 | 44.4 ± 14.8 | Sufficient:  80.5%  (*n* = 58) Insufficient: 15.3%  (n = 11)  Deficient:  4.2%  (*n* = 3) | Average 4 hours training/day. Indoor group lower 25(OH)D than both outdoor/mixed (*p* = 0.0001). 14 out of 15 insufficient/deficient athletes were in indoor group. Only one outdoor athlete was insufficient. |

**Table S1.** *Cont.*

| **Author** | **Sport** | **Subjects/Methods Description** | **Season** | **Location** | **Reference Ranges (ng/mL)** | **Average 25(OH)D (ng/mL)** | **Vitamin D Status** | **Observations** |
| --- | --- | --- | --- | --- | --- | --- | --- | --- |
| Pollock et al.,  2012 [19] | Track & Field | 63 elite athletes training High Performance Athletics Centres  (24 ± 4.2 years; 20 indoor, 43 outdoor) | December 2008–August 2009 | UK (51° N–54° N) | Sufficient: >30 Insufficient: 20–30 Deficient: <20 | 31.5 ± 15.1 | Sufficient:  52% (*n* = 33)  Insufficient:  29% (*n* = 18)  Deficient:  19% (*n* = 12) | Outdoor athletes had higher 25(OH)D than indoor athletes  (*p* = 0.016), but this did not account for month of blood draw. Overall, athletes had higher concentrations in the summer than winter (35.4 ± 15.8 vs.  25.2 ± 11.8, *p* = 0.005). |
| Willis et al.,  2012 [20] | Runners | 19 endurance athletes; Male: (*n* = 9), 27.4 ± 9.4 years;  68.8 ± 3.9 kg; Female: (*n* = 10), 29.1 ± 7.5 years, 56.2 ± 4.9 kg | Entire year | Baton Rouge, LA (30° N) | Sufficient: >32 Insufficient: 20–32 Deficient: <20 | Male:  33.8 ± 14.0 Female:  43.1 ± 19.3 | Sufficient:  47%% (*n* = 9)  Insufficient:  42% (*n* = 8)  Deficient:  11% (*n* = 2) | Inverse relationship between TNF-α and vitamin D (*p* < 0.001). |
| Ducher et al.,  2011 [21] | Ballet | 16 male ballet dancers, 16.0 ± 0.7 years; 61.6 ± 3.9 kg | July  (Winter) | Melbourne, AU (37° S) | Sufficient: >30 Insufficient: 20–30 Deficient: <20 | 20.2  (8.3–37.7) | Sufficient:  19% (*n* = 3)  Insufficient:  25% (*n* = 4)  Deficient:  56% (*n* = 9) | Over half of participants were classified as deficient according to Endocrine Society guidelines. Researchers defined “normal” as concentrations >20 ng/mL (IOM guidelines), however category frequencies presented here correspond to Endocrine Society guidelines. |

**Table S1.** *Cont.*

| **Author** | **Sport** | **Subjects/Methods Description** | **Season** | **Location** | **Reference Ranges (ng/mL)** | **Average 25(OH)D (ng/mL)** | **Vitamin D Status** | **Observations** |
| --- | --- | --- | --- | --- | --- | --- | --- | --- |
| Bescos et al.,  2011 [22] | Basketball (Professional) | 21 male, 25 ± 4.3 years;  93.7 ± 10.4 kg,  9.2 ± 2.6 BF% | March & April | Barcelona, Spain (41° N) | Sufficient: >20 Insufficient: 12–20 Deficient: <12 | 19.2 ± 8.7 | Sufficient:  43% (*n* = 9)  Insufficient:  48% (*n* = 10)  Deficient:  9.5% (*n* = 2) | Dietary vitamin D intake was 139 ± 78 IU/day. 25(OH)D concentrations positively correlated with dietary vitamin D intake (*p* < 0.001). African American players had lower concentrations than Caucasian players  (9.5 ± 3.0 vs.  22.1 ± 6.6 ng/mL). |
| Gibson et al.,  2011 [23] | Soccer (Canadian junior athletes) | 28 female, 15.7 ± 0.7 years; BMI: 22.7 ± 2.7 kg/m^2^ | Spring | British Columbia, Canada (53° N) | Not defined | 75.4 ± 18.5 | Not Reported | Average dietary intakes vitamin D:  (163.3 ± 94.7 IU/day), calcium: (931 ± 351.1 mg/day). Participants taking supplements: MVI (*n* = 2), Fatty acid supplementation (*n* = 1), herbal cold/flu remedy (*n* = 1). |

**Table S1.** *Cont.*

| **Author** | **Sport** | **Subjects/Methods Description** | **Season** | **Location** | **Reference Ranges (ng/mL)** | **Average 25(OH)D (ng/mL)** | **Vitamin D Status** | **Observations** |
| --- | --- | --- | --- | --- | --- | --- | --- | --- |
| Halliday et al.,  2011 [24] | Multiple collegiate sports | Fall (*n* = 41);  M: (*n* = 18) 20.1 ± 1.9 years, 88.0 ± 19.6 kg;  F: (*n* = 23) 19.9 ± 1.5 years, 59.6 ± 10.2 kg  Winter (*n* = 25)  Spring (*n* = 25, *n* = 21  for DXA) | Sept/Oct (Fall), Feb/Mar (Winter), Apr/May (Spring) | Laramie, WY (41.3° N) | Sufficiency: >32 Insufficient: 20–32 Deficient: <20 | Fall:  49 ± 6.6 Winter:  30.5 ± 9.4 Spring:  41.9 ± 14.6 | Sufficient: Fall:  12.2% (*n* = 5) Winter:  21.2% (*n* = 7) Spring:  44.0% (*n* = 11) Insufficient: Fall:  9.8% (*n* = 4) Winter:  60.6% (*n* = 20) Spring:  16% (*n* = 4) Deficient: Fall:  2.4% (*n* = 1) Winter: 3.0% (*n* = 1) Spring: 4.0% (*n* = 1) | Average dietary vitamin D Intake (IU/day):  Fall: 242 ± 161,  Winter: 282 ± 206,  Spring: 204 ± 171.  Vitamin D significantly higher in outdoor athletes compared to indoor in the fall but not the winter or spring. Vitamin D not correlated with bone density. Frequency of injury not related to vitamin D. Injury frequency negatively correlated with total BMD. |
| Morton et al.,  2011 [25] | Soccer (Professional) | 20 soccer players  (2 dark skinned),  26 ± 4 years; 79.5 ± 7.5 kg | August–December | UK (53° N) | Adequate: ≥30 Insufficient: 20–29 Deficient: <20 | August: 41.8 ± 8.5 (27.2–60.5) December: 20.4 ± 7.6 (8.8–34.5) | Only graph representation | In December, 65% < 20 ng/mL. No correlation between body fat and vitamin D status. 2 dark skinned players had the lowest levels in both summer and winter  (Aug: 28.0, 27.2; Dec: 8.8,  15.6 ng/mL). Significant decrease in 25(OH)D from Aug–Dec (*p* < 0.001).  All 20 players saw decrease. Average decrease was  21.1 ± 6.0 ng/mL  (51% ± 15%, 27%–71%). |

**Table S1.** *Cont.*

| **Author** | **Sport** | **Subjects/Methods Description** | **Season** | **Location** | **Reference Ranges (ng/mL)** | **Average 25(OH)D (ng/mL)** | **Vitamin D Status** | **Observations** |
| --- | --- | --- | --- | --- | --- | --- | --- | --- |
| Storlie, et al.,  2011 [26] | Football, cross country, track & field, rugby | 27 Division II athletes,  18–33 years. Athletes were supplemented with 1000 IU of vitamin D mouth spray for 12 weeks | October and January | Central  (46.9° N) | High: >100 Optimal: 40–80 Sufficient: 32–40  Insufficient <32  Deficient <20 | Fall:  127.7 ± 46.6  Winter:  98.5 ± 37.8 | Sufficient: Fall:  70% (*n* = 19)  Winter:  67% (*n* = 18)  Insufficient:  Fall:  25% (*n* = 7)  Winter:  30% (*n* = 8)  Deficient: Fall:  3.7% (*n* = 1)  Winter: 3.7% (*n* = 1) | Average vitamin D intake in fall  (197 ± 32.24 IU/day) and in winter (340 ± 34.85 IU/day). Significant decreases in vitamin D concentrations from fall to winter. No significant correlation between vitamin D and BF%. After 12 weeks, no significant diff. in VITD group vs. CON group. VIT D group had smaller seasonal decrease than CON group. |
| Constantini et al.,  2010 [27] | Multiple sports and dancers | 98 trained athletes and dancers, 14.7 ± 3.0 years; Male: (*n* = 52), Female: (*n* = 46). Vitamin D from 49 obtained during summer months | Winter (Novemner–April)  Summer  (May–October) | Israel (31.8° N) | Sufficient: >30 Insufficient <30 Deficient: <20 | 25 ± 8.3 | Sufficient:  27% (*n* = 26) Insufficient:  48% (*n* = 47)  Deficient:  26% (*n* = 25) | 10/21 outdoor athletes were insufficient, 62/77 of indoor athletes. Overall 73% had concentrations <30 ng/mL. |
| Lovell et al.,  2008 [28] | Gymnastics | 18 female gymnasts,  10–17 years | May (End of Fall) | Canberra, Australia (35.3° N) | Sufficient: >30  Insufficient: 20–30  Deficient: <20 | 22.4  (11.6–33.7) | Sufficient:  16.7% (*n* = 3)  Insufficient:  50% (*n* = 9)  Deficient: <20: 27.8% (*n* = 5)  <12: 5.5% (*n* = 1) | 83.3% of gymnasts did not meet adequate vitamin D concentrations. Over past 12 months  13 gymnasts reported a bony stress fracture diagnosed by clinical examination. 13 had dietary calcium intakes below rec amount  of 1000 mg/day  (range 240–1740 mg). |

Abbreviations

| BMI | body mass index |
| --- | --- |
| DXA | dual X-ray absorptiometry |
| URTI | upper respiratory tract infection |
| PTH | parathyroid hormone |
| IU | international units |
| UVR | ultra-violet radiation |
| IOM | Institute of Medicine |
| XC | cross country |
| MVI | multi-vitamin |

References

1. He, C.; Fraser, W.D.; Tang, J.; Brown, K.; Renwick, S.; Rudland-Thomas, J.; Gleeson, M. The effect of 14 weeks of vitamin D 3 supplementation on antimicrobial peptides and proteins in athletes. *J. Sports Sci.* **2016**, *34*, 67–74.
2. Beck, K.L.; Mitchell, S.; Foskett, A.; Conlon, C.A.; von Hurst, P.R. Dietary Intake, Anthropometric Characteristics, and Iron and Vitamin D Status of Female Adolescent Ballet Dancers Living in New Zealand. *Int. J. Sport Nutr. Exerc. Metab.* **2015**, *25*, 335–343.
3. Fitzgerald, J.S.; Peterson, B.J.; Wilson, P.B.; Rhodes, G.S.; Ingraham, S.J. Vitamin D Status Is Associated with Adiposity in Male Ice Hockey Players. *Med. Sci. Sports Exerc.* **2015**, *47*, 655–661.
4. Heller, J.E.; Thomas, J.J.; Hollis, B.W.; Larson-Meyer, D.E. Relation between vitamin D status and body composition in collegiate athletes. *Int. J. Sport Nutr. Exerc. Metab.* **2015**, *25*, 128–135.
5. Maroon, J.C.; Mathyssek, C.M.; Bost, J.W.; Amos, A.; Winkelman, R.; Yates, A.P.; Duca, M.A.; Norwig, J.A. Vitamin D profile in National Football League players. *Am. J. Sports Med.* **2015**, *43*, 1241–1245.
6. Farrokhyar, F.; Tabasinejad, R.; Dao, D.; Peterson, D.; Ayeni, O.; Hadioonzadeh, R.; Bhandari, M. Prevalence of vitamin D inadequacy in athletes: A systematic-review and meta-analysis. *Sports Med.* **2015**, *45*, 365–378.
7. Koundourakis, N.E., Androulakis, N.E., Malliaraki, N.; Margioris, A.N. Vitamin D and Exercise Performance in Professional Soccer Players. *PLoS ONE* **2014**, *9*, 1–6.
8. Valtueña, J.; Dominguez, D.; Til, L.; González-Gross, M.; Drobnic, F.; Valtueña, J. High prevalence of vitamin D insufficiency among elite Spanish athletes the importance of outdoor training adaptation.
   *Nutr. Hosp.* **2014**, *30*, 124–131.
9. Villacis, D.; Yi, A.; Jahn, R.; Kephart, C.J.; Charlton, T.; Gamradt, S.C.; Romano, R.; Tibone, J.E.; Hatch, G.F., 3rd. Prevalence of Abnormal Vitamin D Levels Among Division I NCAA Athletes. *Sports Health* **2014**, *6*,
   340–347.
10. Close, G.L.; Russell, J.; Cobley, J.N.; Owens, D.J.; Wilson, G.; Gregson, W.; Fraser, W.D.; Morton, J.P. Assessment of vitamin D concentration in non-supplemented professional athletes and healthy adults during the winter months in the UK: Implications for skeletal muscle function. *J. Sports Sci.* **2013**, *31*,
    344–353.
11. Close, G.L; Leckey, J.; Patterson, M.; Bradley, W.; Owens, D.J.; Fraser, W.D.; Morton, J.P. The effects of vitamin D(3) supplementation on serum total 25[OH]D concentration and physical performance:
    A randomised dose-response study. *Br. J. Sports Med.* **2013**, *47*, 692–696.
12. He, C.-S. Influence of vitamin D status on respiratory infection incidence and immune function during 4 months of winter training in endurance sport athletes. *Exerc. Immunol. Rev.* **2013**, *19*, 1077–5552.
13. Kopec, A.; Solarz, K.; Majda, F.; Slowinska-Lisowska, M.; Medras, M. An evaluation of the levels of vitamin d and bone turnover markers after the summer and winter periods in polish professional soccer players*.
    J. Hum. Kinet.* **2013**, *38*, 135–140.
14. Magee, P.J.; Pourshahidi, L.K.; Wallace, J.W.; Cleary, J.; Conway, J.; Harney, E.; Madigan, S.M. Vitamin D status and supplementation in elite Irish athletes. *Int.* *J. Sport Nutr. Exerc. Metab.* **2013**, *23*, 441–448.
15. Wilson, G.; Fraser, W.D.; Sharma, A.; Eubank, M.; Drust, B.; Morton, J.P.; Close, G.L. Markers of Bone Health, Renal Function, Liver Function, Anthropometry and Perception of Mood: A Comparison between Flat and National Hunt Jockeys. *Int.* *J. Sports Med.* **2013**, *34*, 453–459.
16. Wolman, R.; Wyon, M.A.; Koutedakis, Y.; Nevill, A.M.; Eastell, R.; Allen, N. Vitamin D status in professional ballet dancers: Winter vs. summer. *J. Sci. Med. Sport* **2013**, *16*, 388–391.
17. Galan, F.; Ribas, J.; Sánchez-Martinez, A; Calero, T.; Sánchez, A.B.; Muñoz, A. Serum 25-hydroxyvitamin D in early autumn to ensure vitamin D sufficiency in mid-winter in professional football players. *Clin. Nutr.* **2012**, *31*, 132–136.
18. Peeling, P.; Fulton, S.K.; Binnie, M.; Goodman, C. Training Environment and Vitamin D Status in Athletes. *Int.* *J. Sports Med.* **2013**, *34*, 248–252.
19. Pollock, N.; Dijkstra, P.; Chakraverty, R.; Hamilton, B. Low 25(OH) vitamin D concentrations in international UK track and field athletes. *S. Afr.* *J. Sports Med.* **2012**, *24*, 55–59.
20. Willis, K.S.; Smith, D.T.; Broughton, K.S.; Larson-Meyer, D.E. Vitamin D status and biomarkers of inflammation in runners. *Open Access J. Sports Med*. **2012**, *3*, 35–42.
21. Ducher, G.; Kukuljan, S.; Hill, B.; Garnham, A.P.; Nowson, C.A.; Kimlin, M.G.; Cook, J. Vitamin D status and musculoskeletal health in adolescent male ballet dancers a pilot study. *J. Dance Med. Sci.* **2011**, *15*, 99–107.
22. Bescos Garcia, R.; Rodriguez Guisado, F.A. Low levels of vitamin D in professional basketball players after wintertime: Relationship with dietary intake of vitamin D and calcium. *Nutr. Hosp.* **2011**, *26*, 945–951.
23. Gibson, J.C.; Stuart-Hill, L.; Martin, S.; Gaul, C. Nutrition status of junior elite Canadian female soccer athletes. *Int. J. Sport Nutr. Exerc. Metab.* **2011**, *21*, 507–514.
24. Halliday, T.; Peterson, N.; Thomas, J.; Kleppinger, K.; Hollis, B.; Larson-Meyer, D. Vitamin D Status Relative to Diet, Lifestyle, Injury and Illness in College Athletes. *Med. Sci. Sports Exerc.* **2011**, *42*, 335–343.
25. Morton, J.P.; Iqbal, Z.; Drust, B.; Burgess, D.; Close, G.L.; Brukner, P.D. Seasonal variation in vitamin D status in professional soccer players of the English Premier League. *Appl. Physiol. Nutr. Metab.* **2012**, *37*, 798–802.
26. Storlie, D.M.; Pritchett, K.; Pritchett, R.; Cashman, L. 12-week vitamin d supplementation trial does not significantly influence seasonal 25(OH)D status in male collegiate athletes. *Int. J. Health Nutr.* **2011**, *2*, 8–13.
27. Constantini, N.; Arieli, R.; Chodick, G.; Dubnov-Raz, G. High prevalence of vitamin D insufficiency in athletes and dancers. *Clin. J. Sport Med.* **2010**, *20*, 368–371.
28. Lovell, G. Vitamin D status of females in an elite gymnastics program. *Clin. J. Sport Med.* **2008**, *18*, 159–161.
